# Improved relapse-free survival on aromatase inhibitors in breast cancer is associated with interaction between oestrogen receptor-α and progesterone receptor-b

**DOI:** 10.1038/s41416-018-0331-3

**Published:** 2018-11-09

**Authors:** Cameron E. Snell, Madeline Gough, Cheng Liu, Kathryn Middleton, Christopher Pyke, Catherine Shannon, Natasha Woodward, Theresa E. Hickey, Jane E. Armes, Wayne D. Tilley

**Affiliations:** 1 Cancer Pathology Research Group, Mater Research Institute—The University of Queensland, Translational Research Institute, Woolloongabba, QLD 4102 Australia; 2 Department of Anatomical Pathology, Mater Pathology, Mater Hospital Brisbane, South Brisbane, QLD 4101 Australia; 3 Department of Medical Oncology, Mater Hospital Brisbane, South Brisbane, QLD 4101 Australia; 4 Department of Breast and Endocrine Surgery, Mater Hospital Brisbane, South Brisbane, QLD 4101 Australia; 5 Mater Research Institute—The University of Queensland, Translational Research Institute, Woolloongabba, QLD 4102 Australia; 6 Dame Roma Mitchell Cancer Research Laboratories, Adelaide Medical School, University of Adelaide, Adelaide, SA 5000 Australia

**Keywords:** Breast cancer, Predictive markers, Breast cancer, Predictive markers, Hormone receptors

## Abstract

**Background:**

Recent pre-clinical studies indicate that activated progesterone receptor (PR) (particularly the PR-B isoform) binds to oestrogen receptor-α (ER) and reprogrammes transcription toward better breast cancer outcomes. We investigated whether ER and PR-B interactions were present in breast tumours and associated with clinical parameters including response to aromatase inhibitors.

**Methods:**

We developed a proximity ligation assay to detect ER and PR-B (ER:PR-B) interactions in formalin-fixed paraffin-embedded tissues. The assay was validated in a cell line and patient-derived breast cancer explants and applied to a cohort of 229 patients with ER-positive and HER2-negative breast cancer with axillary nodal disease.

**Results:**

Higher frequency of ER:PR-B interaction correlated with increasing patient age, lower tumour grade and mitotic index. A low frequency of ER:PR-B interaction was associated with higher risk of relapse. In multivariate analysis, ER:PR-B interaction frequency was an independent predictive factor for relapse, whereas PR expression was not. In subset analysis, low frequency of ER:PR-B interaction was predictive of relapse on adjuvant aromatase inhibitor (HR 4.831, *p* = 0.001), but not on tamoxifen (HR 1.043, *p* = 0.939).

**Conclusions:**

This study demonstrates that ER:PR-B interactions have utility in predicting patient response to adjuvant AI therapy.

## Background

Approximately 80% of breast cancers express oestrogen receptor-α (ER)^1,2^ and are considered to be driven by the trophic effects of oestrogen. Expression of ER by immunohistochemistry remains the only clinical biomarker predictive of benefit to adjuvant anti-oestrogen therapies, which include two broad classes of drugs; non-steroidal aromatase inhibitors (AIs) and the selective ER modulator, tamoxifen. Progesterone receptors (PR, comprising A and B isoforms) are upregulated in response to ER signalling in normal and malignant breast tissues.^3^ Antibodies used to detect PR for clinical and investigative purposes largely detect both PR isoforms and using these, PR has been established as a biomarker of good prognosis in breast cancer.^4^ Higher levels of PR expression are associated with a good response to tamoxifen,^4,5^ which until the development of aromatase inhibitors (AIs) was the major first-line adjuvant endocrine therapy for all cases of ER-positive (ER+) breast cancer. Currently, tamoxifen is mainly prescribed to pre-menopausal women and AIs to postmenopausal women, with some exceptions.^6–8^ In general, AIs may confer a survival advantage compared to tamoxifen.^9^ However, PR expression does not predict therapeutic benefit of AIs.^6–8^

Pre-clinical studies have shown that ER and PR form a physical interaction in the presence of their cognate hormones and that this activity may promote better disease outcomes.^10–14^ In the presence of oestrogen and a progestogen, including endogenous progesterone (P4), PR alters the interaction between ER and chromatin to change the transcriptional output of ER+ breast cancer cells.^12–14^ Ligand-activated PR redirects ER chromatin binding to sites enriched for progesterone response motifs^12^ and distal enhancers enriched for BRCA1 motifs.^13^ Moreover, expression of a gene signature associated with PR-mediated reprogramming of ER binding is associated with good prognosis in primary breast cancer cohorts.^12^ Consistent with these findings, progestogen treatment inhibits oestrogen-dependent growth in various preclinical models of breast cancer (e.g. breast cancer cell lines, ex-vivo culture of clinical breast cancer tissues and patient-derived xenografts).^12–14^

Two progesterone receptor isoforms, PR-A and PR-B, are transcribed from a single gene, the PGR.^15^ The two isoforms are identical apart from an additional 165 amino acids present in the N-terminus of PR-B. In the presence of both ER and PR agonist ligands, immunoprecipitation of ER shows a specific increase in PR-B interaction in the ER+, PR+ breast cancer cell line T47D.^13^ However, in the presence of activated ER, unliganded PR promotes expression of a subset of ER target genes and enhances proliferation,^11^ highlighting the importance of ligand activation of PR. Up to 29% of ER+ breast cancers have a heterozygous or homozygous deletion of PGR, which occurs more often in the luminal B subtype of breast cancers.^12^ Therefore, it is not surprising that the luminal B breast cancer subtype is associated with a higher proliferation rate and poorer prognosis than luminal A cancers.^16^ Loss of PGR is a mechanism by which ER+ tumours may evade the antagonistic effect of PR signalling on ER-mediated oncogenesis. In support of this, loss of PGR in ER+ cancers is associated with poor prognosis.^12^ In ER+PR+ tumours, lack of adequate PR activation could also feasibly be a cause of unrestrained ER activity.

The ratio of PR-A to PR-B has been investigated by immunoblot analysis and varies between breast cancers.^17–20^ Patients with PR+ tumours that have a lower proportion of PR-B have a worse prognosis and are more likely to relapse on tamoxifen,^18^ while tumours with a higher proportion of PR-A responded to the antiprogesterone mifepristone in ex vivo models.^19^ Patients whose tumours expressed a gene signature associated with a high PR-A to PR-B ratio also have a poorer survival outcome.^21^ The two isoforms differentially reprogramme ER-binding: PR-B predominantly acts to redistribute ER genomic recruitment while PR-A predominantly inhibits ER chromatin binding.^21^ In T47D cells engineered to express a single isoform, only PR-B decreased oestrogen-induced invasion.^21^ These findings suggest PR-mediated reprogramming of ER is dependent on PR isoform-specific expression.

Oestrogen is present at sufficient levels in post-menopausal women to promote ER+ breast cancer and these patients benefit from treatment with aromatase inhibitors.^7^ The majority of oestrogen production in postmenopausal women occurs in peripheral tissues that express aromatase, including the breast.^22^ Since circulating progesterone is present at very low levels in postmenopausal women,^23^ exogenous treatment with a PR agonist may be a therapeutic strategy to benefit patients with ER+ breast cancer by exploiting cross-talk between ER and PR.^24^ In advanced ER+ breast cancer, trials of progestins such as megesterol acetate or medroxyprogesterone acetate have consistently shown significant clinical benefit, including in women who had previously relapsed on either an AI^25^ or tamoxifen.^26,27^ Clinical trials are underway to test efficacy of progestogens in the neoadjuvant setting.^28^ Although the abovementioned studies propose that induction of ER:PR-B interaction would be therapeutically beneficial, such interactions have not yet been shown to occur in clinical specimens. The aim of our study was to validate a proximity ligation assay (PLA) to detect an interaction between ER and PR-B (ER:PR-B) in formalin-fixed, paraffin embedded (FFPE) tissues and investigate whether this interaction was predictive of relapse in a cohort of women with ER+ breast cancer treated with adjuvant endocrine therapy.

## Methods

### Cell culture

The T47D breast cancer cell line was acquired from the ATCC and cultured in DMEM supplemented with 10% FBS. The cells were regularly tested for mycoplasma infection. To stimulate ER and PR interactions, the cells were first cultured in phenol red-free DMEM supplemented with charcoal stripped FBS (Gibco, no. 12676011) for 48 h, then the media was supplemented with vehicle (ethanol), oestradiol (E_2_) (Sigma, no. E2758), progesterone (Sigma, no. P6149) or the combination of E_2_ and progesterone both at a final concentration of 10 nM for 24 h. Cells were mechanically lifted, fixed in 10% neutral buffer formalin for 24 h then resuspended in 2% molten agarose dissolved in 10% formalin. The cells and agarose suspension were centrifuged to form a pellet. Cell pellets were processed in tissue cassettes at Mater Pathology as per clinical specimens.

### Patient-derived tumour explants (PDEs)

Tumour samples were obtained following informed consent from women undergoing surgery for breast cancer at the Burnside War Memorial Hospital, Adelaide. This study was approved by the University of Adelaide Human Research Ethics Committee (approval numbers: H-065-2005; H-169-2011). Excised tissue samples were cultured ex vivo as previously described.^12,29^ Briefly, PDEs were cultured on gelatine sponges for 36 h then treated with the following conditions: vehicle (ethanol), E_2_ (10 nM), a synthetic progestin R5020 (10 nM) or the combination of E_2_ and R5020 (both at 10 nM) with treatment for 48 h. Explants were fixed in 10% neutral buffered formalin overnight and processed as per clinical specimens.

### Proximity-ligation assays

The proximity-ligation assay (PLA) can be used to detect proteins, interactions and modifications with high sensitivity and specificity.^30^ PLA requires protein recognition by pairs of antibody conjugates and improves specificity of protein detection over immunohistochemical assays.^31^ FFPE tissues were sectioned at 6 µM, deparaffinised and antigen retrieved in citrate buffer at pH 6 using a Decloaking Chamber (Biocare Medical). Sections were blocked, and primary antibodies were diluted in antibody diluent (Roche, no. 251-018) and incubated overnight at 4 °C. To detect ER and PR-B interactions, antibodies from two different species were used; monoclonal rabbit anti-ER (Thermo Scientific, clone SP1) and monoclonal mouse anti-PR (Sigma, clone 3E11 – raised against an immunogen specific to the amino-terminus of the PR-B isoform), both used at 1:100 dilution. For expression of PR-B, the same mouse anti-PR-B was incubated with rabbit anti-PR (Ventana, clone 1E2, detecting both PR A and B isoforms^32^). This was followed by incubation with the PLA-probes Duolink in Situ PLA Probe Anti-Mouse PLUS (Sigma, no. DUO92001-100RXN) and Anti-Rabbit MINUS (Sigma, no. DUO92005-100RXN) for 60 min at 37 °C in a pre-heated humidity chamber. Ligation took place for 30 min and amplification for 120 min at 37 °C using Duolink in Situ Detection reagents brightfield (Sigma, no. DUO92012-100RXN). To detect the rolling circle amplification product, horse radish peroxidase-conjugated probe was incubated for 60 min at room temperature and substrate solution was applied for 10 min also at room temperature. Slides were counterstained with haematoxylin. Staining was independently scored by two breast histopathologists (CS, CL) by counting the number of signals per nucleus in 20 cells in the areas of tumour with greatest numbers of signals, a similar method to that used to score HER2 detected by in situ hybridisation assays. Scores were averaged to determine a final score. The interactions detected by this assay are referred to as “ER:PR-B” and the signals detected by the PR-B PLA are referred to as “PR-B”.

### Immunohistochemistry

ER and PR immunohistochemistry was performed with anti-ER (Ventana, clone SP1) and anti-PR (Ventana, clone 1E2) using the Ventana BenchMark ULTRA automated slide stainer (Roche). The Ventana anti-PR antibody clone 1E2 used widely by diagnostic pathology laboratories recognises both the PR A and B isoforms.^32^ ER and PR immunohistochemistry was scored by two breast histopathologists (CS, CL) using the ‘Allred score’ on a scale of 0–8.^33^ In brief, the proportion of positive cells was evaluated as 0 = no positive cells, 1 = 0– <1% positive cells, 2 = 1– <10% positive cells, 3 = 10– <33% positive cells, 4 = 33– <66% positive cells and 5 = >66% positive cells. Additionally, the average intensity of staining was scored as 0 = negative, 1 = weak, 2 = moderate or 3 = strong. The intensity and proportion scores were added to obtain the ‘Allred score’. A cut-off of >2 was considered positive (weak positive staining in >1% of tumour cell nuclei) which is the cut-off used clinically.^34^

### Study population

A cohort comprising a consecutive series of 229 patients who had surgery with curative intent for ER+, human epidermal growth factor receptor 2-negative (HER2-negative) node-positive breast cancer (supplementary material Table S1) was analysed. All patients had lymph node metastatic deposits of at least 2.0 mm in size resected with curative intent (at least N1, all patients were stage II and III^35^). Patients had their tumours resected at the Mater Hospital Brisbane between January 2005 and December 2014. No patients had endocrine therapy prior to surgery. HER2 negativity was defined by negative immunohistochemistry and lacking amplification by in situ hybridisation of the *ERBB2* gene. Recommendations for adjuvant treatment were made at the breast multidisciplinary meeting according to international guidelines and treatment decisions were made by patients in conjunction with their treating specialists. The median age of patients was 54 years at resection (range 27–88 years) and 71.2% were postmenopausal. 86.0% of patients had adjuvant chemotherapy, 81.7% adjuvant radiation and 94.8% adjuvant endocrine therapy. Of those that had endocrine therapy, 69.6% were treated with an aromatase inhibitor and 29.0% with tamoxifen. The median follow-up time was 5.1 years (range 0.9–11.3 years). Relapse was defined as either clinically or radiologically detected locoregional or distant metastatic disease. Relapse occurred in 48 patients (21%) and the mean estimated relapse-free survival time was 8.8 years (SD 3.7 years). All patients were recommended adjuvant endocrine therapy post-surgery and patients were considered not to have taken adjuvant endocrine therapy if they took a total of <2 months treatment. The use of clinical information and tumour blocks was approved by the Mater Health Services Human Research Ethics Committee (approval number: HREC/15/MHS/123). Cores of the primary tumour from each patient were assembled into tissue microarrays (four cores per patient, each measuring 1.0 mm in diameter)^36^ using a semi-automated arrayer (Beecher Instruments). Four 1.0 mm cores have previously demonstrated spatial heterogeneity for ER in only 2% of cases and PR in 7% of cases.^37^ The study was designed to meet the REMARK guidelines for reporting tumour marker prognostic studies.^38^

### Statistical design and analysis

Statistical analysis was performed using SPSS V.22.0 (IBM) and GraphPad Prism V.7.03 (GraphPad Software, Inc). Correlations between ER:PR-B interactions, PR-B expression and clinical and pathological factors were determined using the 2-tailed Spearman’s rank correlation coefficient (*r*) as ER:PR-B interactions were not normally distributed. The Mann–Whitney U test was used to compare test whether number of ER:PR-B interactions differed between PR− and PR+ groups. Receiver operating characteristic (ROC) curves were used to determine the optimum cut-off of signals per cell with respect to relapse. Relapse-free survival analyses were carried out using Kaplan–Meier curves and significance determined by log-rank test. Univariate and multivariate Cox regression analyses were used to determine significant dependent and independent variables. Factors significant in the univariate analysis were included in the multivariate analysis. Associations for 2 × 2 tables were carried out using a Fisher’s exact test, due to small numbers in some subgroups. The 3 × 2 table for association of tumour histologic type was tested for significance using a Fisher’s exact test. All other 3 × 2 tables and 4 × 2 tables were tested for significance using the Cochran-Armitage test for trend, due to the variables being ordinal.

## Results

### Visualisation of ER and PR interactions in FFPE tissue

**Fig. 1:**
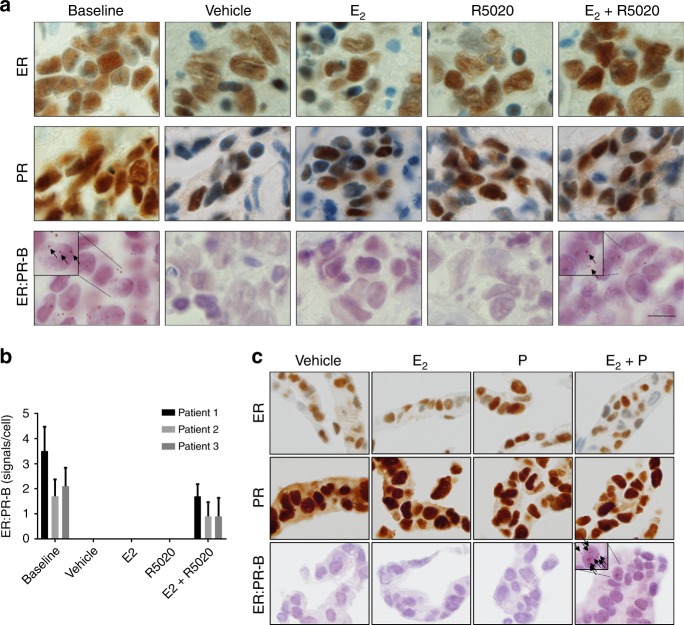
Development of a proximity ligation assay to detect interactions between the oestrogen receptor-α and progesterone receptor-B (ER:PR-B) in patient derived breast cancer explants and T47D cells. **a** Breast cancer explants were treated with either Vehicle, oestradiol (E_2_), R5020 or the combination of E_2_ and R5020. Explants were immunohistochemically stained for the oestrogen receptor-α (ER), progesterone receptor (PR) and the ER:PR-B proximity ligation assay (PLA). Scale bar (bottom right, applies to all images) is 10 µM. **b** Bar graph indicates mean number of ER:PR-B interactions and standard deviation per cell for three explants with each condition. **c** T47D cells were treated with either Vehicle, E_2_, progesterone (P) or the combination of E_2_ and P. Cells were immunohistochemically stained for ER, PR and stained using the ER:PR-B PLA

We applied an ER:PR-B proximity ligation assay (PLA) to three ER+, PR+ PDEs treated with vehicle, E_2_, the synthetic progestin R5020 or the combination of E_2_ and R5020. An interaction between ER and PR-B was observed in all three of the untreated (baseline) PDEs to a varying degree (Fig. 1a, b). In treated explant tissues, ER:PR-B interactions were only detected in the presence of both hormones (E_2_ + R5020) (Fig. 1a, b). There was no detectable difference in levels of ER and PR among explants by immunohistochemistry in the four treatment groups for any case. In T47D cells, ER and PR have been previously shown to interact in the presence of E_2_ and progestogen using co-immunoprecipitation technologies.^10–12^ To show this in situ, T47D cells were treated for 24 h with vehicle, E_2_, progesterone or the combination of E_2_ and progesterone under steroid-depleted conditions. As observed in the treated PDEs, ER:PR-B interactions were only detected in FFPE cell pellets of T47D cells treated with the combination of E2 and progesterone (Fig. 1c). Again, there was no detectable change in ER and PR by immunohistochemistry among treatments. Collectively these data show that ER:PR-B interactions only occur in PDEs when both receptors are acutely ligand-activated.

### Association between ER:PR-B interactions, PR-B expression by PLA and ER and PR immunohistochemistry in breast cancers

**Fig. 2:**
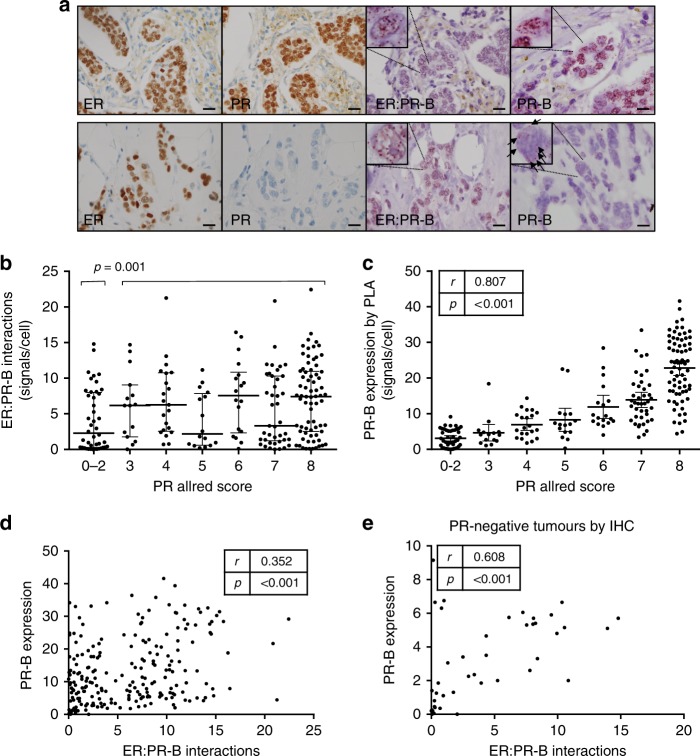
Associations between total PR expression, ER:PR-B interactions and PR-B expression by proximity ligation assay and example photomicrographs. 227 patients with ER+, HER2- breast cancer with paired evaluable expression of PR by immunohistochemistry (Allred score), ER:PR-B interactions and PR-B expression by proximity ligation assay (signals/cell) are represented. **a** Example immunostains for ER, PR and PLA images for two patients. Scale bar is 20 µM. **b** Association between ER:PR-B interactions and PR expression (A/B) by immunohistochemistry. Line indicates median with interquartile range. *P* value is the result of the Mann–Whitney U test. **c** Association between PR-B expression by PLA and PR expression (A/B) by immunohistochemistry. **d** Association between PR-B expression by PLA and ER:PR-B interactions. **e** Association between PR-B expression by PLA and ER:PR-B interactions in 44 patients negative for PR (A/B) by immunohistochemistry

The ER:PR-B PLA was applied to 229 primary tumours arranged in quadruplicate on a TMA and scored by counting the number of signals per tumour cell nucleus. The vast majority of interactions were intranuclear and very occasional cases demonstrated cytoplasmic interactions. All cases had detectable ER by immunohistochemistry at a clinically relevant level (>1% positive tumour nuclei).^34^ There was a significant positive correlation between ER:PR-B interactions and PR expression (*p* = 0.003) (Table 1). Similarly, ER:PR-B interactions and ER expression were positively correlated (*p* = 0.001) (Table 1). In 44 tumours that were negative for PR by immunohistochemistry (Allred score 0–2; representing weak positive staining in less than 1% of tumour cells), the median number of ER:PR-B interactions was 2.28 (inter-quartile range 1.8–10.3), significantly lower than the number of interactions in cases with a PR Allred score of 3 or more (median 6.45 signals per cell, inter-quartile range 0.26–7.99, *p* = 0.001) (Fig. 2b). However, the number of ER:PR-B interactions detected was not absolutely dependent on relative expression of PR by IHC; many cases with high levels of PR expression showed very few detectable ER:PR-B interactions, and conversely there were cases with significant numbers of ER:PR-B interactions in the absence of detectable PR expression by IHC (Fig. 2a).

**Table 1:** Correlations between ER:PR-B interactions (signals/cell) and clinicopathological variables

Variable	Spearman’s *r*	*p*	*N*
Age	0.229	0.001	227
Post-menopausal	0.133	0.045	227
T-stage	0.056	0.405	227
N-stage	–0.077	0.247	227
Grade	–0.144	0.030	227
Mitotic score	–0.198	0.003	227
Multiple tumours	–0.039	0.555	227
Mastectomy	–0.056	0.402	227
Axillary clearance	−0.020	0.769	227
Adjuvant chemotherapy	−0.058	0.382	226
Adjuvant radiation	0.019	0.778	224
Adjuvant endocrine therapy	−0.044	0.511	226
ER expression (allred score)	0.567	0.001	227
PR expression (allred score)	0.199	0.003	227
PR-B expression (PLA signals)	0.352	0.001	227

The *p* value quoted is the result of a 2-tailed Spearman’s correlation. *N* is the number of patients with pairwise non-missing values

We postulated that detection of ER:PR-B interactions in the absence of PR immunostaining was due to the increased sensitivity of the PLA over the immunohistochemical assay for PR. To investigate this, we developed a PLA to determine specific expression of the PR-B isoform (Fig. 2a). PR-B expression was highly correlated with PR (A/B) expression by IHC (*r* *=* 0.807; *p* = < 0.001) (Fig. 2c). There was a significant correlation between PR-B expression and ER:PR-B interactions (*r* *=* 0.352; *p* = < 0.001) (Fig. 2d). In the 44 patients that were negative for PR by IHC, there was significant positive correlation between ER:PR-B interactions and PR-B expression by PLA (*r* *=* 0.608; *p* = < 0.001) (Fig. 2e), demonstrating that detection of PR-B by PLA is more sensitive than detecting PR by IHC.

### Correlations between ER:PR-B interactions and clinical and pathological variables

There was a positive correlation between ER:PR-B interactions and age (*p* = 0.001), with a higher number of interactions in post-menopausal women (Table 1). Higher ER:PR-B interactions were correlated with lower tumour grade (*p* = 0.030) and lower mitotic score (*p* = 0.003). There was no significant correlation with T-stage, N-stage, the presence of multiple tumours, or type of surgical or adjuvant treatment.

### Association of ER and PR immunohistochemistry and ER:PR-B interactions with relapse-free survival

**Fig. 3:**
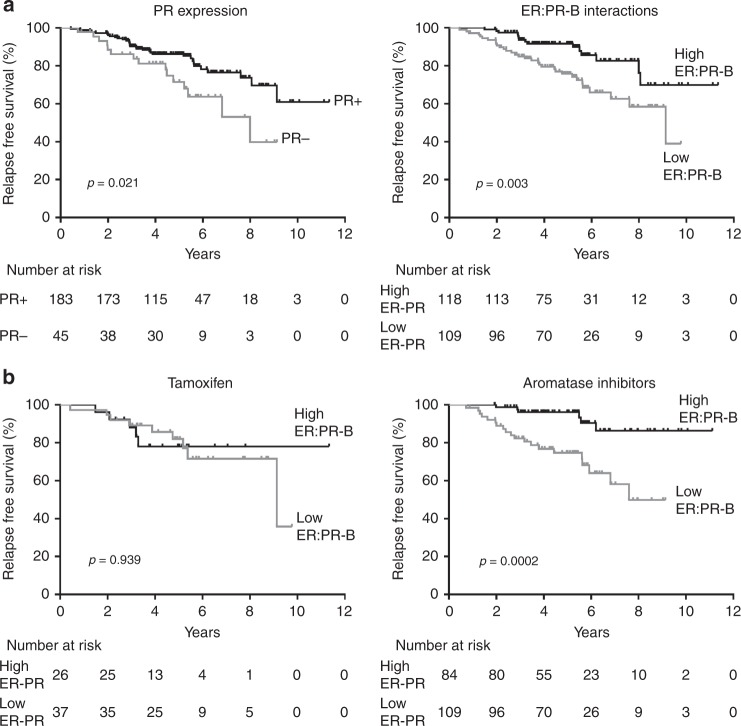
Kaplan–Meier curves by PR expression and ER:PR-B interactions (**a**) and ER:PR-B interactions stratified by type of adjuvant endocrine agent taken (**b**). *P* values quoted are the result of the log-rank test

In the cohort as a whole, absent PR immunohistochemistry was associated with poorer relapse free survival (log-rank *p* = 0.021) (Fig. 3a). ROC curve analysis was used to determine an optimal cut-off for the number of ER:PR-B interactions per cell in patients who had received adjuvant tamoxifen or an AI using relapse status as the dependent variable (supplementary material, Figure S1). In AI-treated patients, the area under the curve was 0.701 (*p* *=* 0.0013) with a cut-off of 5 signals per cell. The sensitivity for detecting relapse was 76.9% and specificity was 63.4%. Similar ROC curve analysis for ER expression (Allred Score) showed no significant association with relapse (supplementary material, Figure S2). When the whole cohort of patients was dichotomised into low (≤5) and high ER:PR-B interactions, those with low ER:PR-B interactions had significantly poorer relapse free survival (log-rank *p* = 0.003) (Fig. 3a).

### Univariate and multivariate Cox regression analyses of variables affecting relapse

In an analysis of the whole cohort of patients, higher pathological T-stage and N-stage were significantly associated with relapse in univariate analysis (Table 2). Patients that underwent chemotherapy and took prescribed endocrine therapy had a significantly reduced risk of relapse. Absent PR expression was significantly associated with relapse (HR 2.028, CI 1.100–3.731, *p* = 0.024) and low levels of ER:PR-B interactions had a higher risk of relapse (HR 2.463, CI 1.333–4.545, *p* = 0.004). There was no significant prognostic effect of age, grade, mitotic score, histologic type, multiple tumours, type of surgery, adjuvant radiotherapy or class of endocrine agent taken.

**Table 2:** Univariate and multivariate Cox regression analysis of clinicopathological factors influencing relapse-free survival in ER+, HER2−, node positive breast cancer patients

Variable	Univariate analysis			Multivariate analysis including ER:PR-B interactions			Multivariate analysis including ER:PR-B interactions and PR-B expression		
	HR	95% CI	*p* Value	HR	95% CI	*p* Value	HR	95% CI	*p* Value
Age	1.002	0.979–1.026	0.835						
T-stage pT2/3/4 vs pT1	3.383	1.214–9.427	**0.020**	3.031	1.063–8.649	**0.038**	3.209	1.128–9.130	**0.029**
N-stage pN2/3 vs pN1	1.999	1.122–3.561	**0.019**	1.623	0.890–2.961	0.114	1.536	0.842–2.802	0.162
Grade 2 vs 1	3.080	0.735–12.897	0.124						
Grade 3 vs 1	1.804	0.862–3.774	0.117						
Mitotic score 2 vs 1	1.139	0.574–2.261	0.710						
Mitotic score 3 vs 1	1.175	0.801–1.724	0.408						
Histology Lobular vs NST	1.448	0.678–3.091	0.339						
Histology Other vs NST	1.433	0.722–2.841	0.304						
Tumour number ≥2 vs 1	1.602	0.735–3.495	0.236						
Mastectomy vs conservation	0.792	0.421–1.488	0.468						
Axillary clearance vs sentinel node only	2.610	0.627–10.853	0.187						
Adjuvant chemotherapy vs not	0.376	0.171–0.827	**0.015**	0.624	0.280–1.391	0.249	0.530	0.236–1.187	0.123
Adjuvant radiation vs not	0.740	0.3501.564	0.431						
Adjuvant endocrine therapy vs not	0.305	0.129–0.720	**0.007**	0.299	0.116–0.767	**0.012**	0.374	0.146–0.954	**0.040**
Aromatase inhibitor vs tamoxifen	0.803	0.3861.669	0.557						
PR expression Negative vs positive	2.028	1.100–3.731	**0.024**	1.543	0.789–3.021	0.205	1.220	0.619–2.404	0.566
PR-B expression Low vs high	3.636	1.543–8.621	**0.003**				2.841	1.134–7.143	**0.026**
ER:PR-B interactions low vs high	2.463	1.333–4.545	**0.004**	2.475	1.297–4.717	**0.006**	2.101	1.092–4.049	**0.026**

Clinical factors significant in the univariate analysis are included in the multivariate model. Bold indicates significant *p* values

ROC analysis was used to determine an optimal cut-off for the number of PR-B signals per cell in patients who had received adjuvant tamoxifen or an AI using relapse status as the dependent variable (supplementary material, Figure S3). Using dichotomised expression with a cut-off of 13.5 signals per cell, sensitivity for detecting relapse was 57.1% and specificity was 92.9% for tamoxifen treated patients and 43.1% and 84.6% respectively for AI-treated patients. Low PR-B expression was significantly associated with relapse (HR 3.636, CI 1.543–8.621, *p* = 0.003).

In a multivariate model that included dichomised levels of ER:PR-B interactions (low ≤5; high >5) and other standard clinical factors significant in univariate analysis, only low levels of ER:PR-B interaction (HR 2.475, CI 1.297–4.717, *p* = 0.006), higher T-stage (HR 3.031, CI 1.063–8.649, *p* = 0.038) and endocrine therapy (HR 0.335, CI 0.121–0.926, *p* = 0.035) were independent prognostic factors associated with relapse (second column, Table 2). Absent PR expression, N-stage and having adjuvant chemotherapy were not significant independent prognostic factors for relapse. In a multivariate model that included both ER:PR-B interactions and PR-B expression, both ER:PR-B interactions and PR-B expression were independent prognostic factors associated with relapse (third column, Table 2).

### Prognostic effect of ER:PR-B interactions and PR-B expression stratified by type of adjuvant endocrine agent

In an exploratory analysis of ER:PR-B interactions stratified by type of endocrine agent, a low frequency of ER:PR-B interaction was associated with relapse in patients taking AIs as adjuvant therapy (log-rank *p* *=* 0.0002), but not with those taking tamoxifen (log-rank *p* = 0.939) (Fig. 3b). This equated to a hazard ratio of 4.831 (CI 1.942–12.048, *p* = 0.001) for patients with low ER:PR-B interactions taking an AI (Table 3). A test for interaction was significant (*p* = 0.031). Patients taking adjuvant endocrine therapy had significant clinical and pathological differences depending on the type of agent (supplementary material, Table S2). Patients on tamoxifen were younger, more likely to be pre-menopausal, had lower T-stage and a higher proportion had tumours with the histology ‘no special type’. In patients taking adjuvant tamoxifen, there was a trend towards a greater proportion of tumours with low levels of ER:PR-B interactions (*p* = 0.051).

**Table 3:** Cox regression analysis of ER:PR-B interactions and PR-B expression influencing relapse-free survival stratified by adjuvant endocrine agent class taken

Endocrine agent	ER:PR-B interaction	Relapse (%)	HR	95% CI	*p* Value
Tamoxifen	Low	9 (24.3)	1.043	0.348–3.135	0.939
	High	5 (19.2)			
Aromatase inhibitor	Low	20 (30.8)	4.831	1.942–12.048	**0.001**
	High	6 (7.1)			
Test for interaction					**0.031**
	**PR-Bexpression**	**Relapse (%)**	**HR**	**95% CI**	***p*** **Value**
Tamoxifen	Low	13 (36.1)	8.929	1.164 – 66.667	**0.035**
	High	1 (3.7)			
Aromatase inhibitor	Low	22 (23.9)	2.874	0.984 – 8.403	0.053
	High	4 (7.0)			
Test for interaction					0.355

Percentages refer to the number of patients with high or low ER:PR-B interactions or PR-B expression that had relapsed on endocrine treatment. Bold indicates significant *p* values

When levels of PR-B were stratified by type of endocrine agent taken, low PR-B expression was only significantly associated with relapse in women taking adjuvant tamoxifen (HR 8.929, CI 1.164–66.667, *p* = 0.035) (Table 3). A test for interaction was not significant (*p* *=* 0.355) There was no significant association between PR expression stratified by type of adjuvant endocrine agent and relapse in patients either taking an AI or tamoxifen (supplementary material, Table S3).

## Discussion

Herein we report the development and application of a novel ER:PR-B interaction assay using PLA that can be used in FFPE breast cancer tissue sections and show that the frequency of these interactions is able to predict response to adjuvant AI therapy. In breast cancer cells in vitro and in treated PDEs, the interaction between ER and PR-B was dependent on ligand-activation of both ER and PR-B by E2 and progestogen, respectively. No interactions were detected in the presence of a single agonist ligand. This finding is consistent with ligand activation of both steroid receptors being required to promote the formation of a functional ER:PR-B complex.^12,13^ In multivariate analysis, we found that PR expression determined by an accredited diagnostic laboratory was not an independent predictive factor for relapse following adjuvant AI therapy, consistent with previous studies.^6–8^ However, low levels of ER:PR-B interactions were predictive of relapse in the AI setting. These findings suggest that ER-PR interactions are a major determinant of the prognostic value of PR expression.

PR-B expression determined by PLA strongly correlated with the levels of total PR by immunohistochemistry, consistent with previous reports.^18,20^ In the 44 cases negative for PR by immunohistochemistry, there was a correlation between PR-B expression determined by PLA and ER:PR-B interactions suggesting the presence of low levels of PR-B in the tumours was sufficient to interact with ER. Lower levels of PR-B were associated with a significantly increased risk of relapse, which in sub-group analysis was limited to patients on adjuvant tamoxifen. This is consistent with previous reports showing lower PR-A to PR-B ratios were associated with poorer survival on tamoxifen.^18,39^ In the cohort analysed as a whole, multivariate analysis demonstrated PR-B expression and ER:PR-B interactions were both independent predictors of relapse, and in a sub-group analysis they differentially predict risk dependent on type of adjuvant therapy. ER:PR-B interactions were not associated with relapse in tamoxifen treated patients. This may be due to the small numbers of tamoxifen-treated patients in this cohort or the mechanism of action of tamoxifen. Tamoxifen directly binds ER and promotes interactions with corepressors,^40^ possibly disrupting interactions with PR-B.

A higher frequency of ER:PR-B interactions was detected in post-menopausal women. Whilst circulating levels of oestrogen and progesterone both dramatically decline with the onset of menopause,^24^ peripheral conversion of circulating androgens to oestrogen increases in many tissues, including the breast.^41,42^ Indeed, tissue levels of oestrogen in the post-menopausal breast are sufficient to promote the development of ER+ breast cancer, which forms the basis of clinical benefit from AIs.^43^ However, peripheral production of progestogens within post-menopausal tissues is not well characterised. Progesterone is detectable in the breast tissue of post-menopausal women with breast cancer using highly sensitive mass-spectrometry methodology and has been recently reported to represent 2.1% of total steroids extracted, about twice the percentage represented by oestrogen (1%).^41^ Since there is no known mechanism of local production of progesterone in breast tissue, the major difference in ER:PR-B interactions between postmenopausal tumours in our study is most likely due to differences in the circulating levels of progesterone and the level of progesterone metabolising enzymes expressed by the tumour or cells in the microenvironment. In both normal and malignant breast tissues, progesterone can be metabolised into 5α-pregnanes and 4-pregnenes by 5α-reductase and 3α-hydroxysteroidoxidoreductase enzymes.^44^ Interestingly, we observed some tumours with detectable ER by immunohistochemistry and PR-B by PLA that showed no evidence of interaction by PLA. This finding likely reflects the degree of interaction between ER and PR-B being more dependent on the availability of agonist ligands than the receptor levels in the individual tumours. Ligand-activated PR-B promotes interaction with ER to reprogram the ER-associated cistrome and induce a transcriptome associated with good clinical outcome^12^; herein we demonstrate that this interaction exists in clinical tissues and is associated with increased relapse-free survival. These findings support the concept currently being tested in clinical trials that promoting ER:PR-B interactions by therapeutic administration of a progestogen may be an effective adjuvant treatment strategy for ER+ breast cancer.^28,45,46^ Indeed, assessment of ER:PR-B interactions using our new assay may be a means of monitoring treatment response in those trials.

Low levels of ER:PR-B interactions were observed in tumours with more aggressive features (higher tumour grade and increased numbers of mitotic figures). This is consistent with ER:PR-B interactions being associated with PR-B reprogramming of ER chromatin binding to promote a transcriptional output associated with tumour-suppressive processes including differentiation and cell death.^12–14^ One variable affecting ER:PR-B interactions in breast tumours is that the PR gene is often lost in ER+ tumours due to deletion^12,47–49^ or its expression is reduced due to hypermethylation of the *PGR* gene locus.^50^ In particular, PR expression typically is lost or reduced in more aggressive luminal B breast cancers,^12,13^ which like all ER+ cancers are more common in postmenopausal women.^16^ In this situation, PR-B reprograming of ER signalling would not occur, leading to maintenance of a growth stimulatory state and a poor disease outcome. In our study we do not know the status of the PR gene in tumours that did not have detectable PR by immunohistochemistry. However, we found that some of these tumours had detectable ER:PR-B interactions and PR-B expression by PLA, indicating an intact PR gene. In current trials involving progestogen therapy, tumours that lack PR by IHC would be deemed ineligible for treatment. Use of our PR-B and ER:PR-B PLA could represent a more sensitive assay to determine eligibility criteria for such trials.

Patients with PR-negative tumours consistently have a poorer prognosis than those with PR+ tumours,^51^ regardless of adjuvant endocrine agent taken.^6–8^ We also found that patients with tumours that were PR-negative by immunohistochemistry had a poorer prognosis, but the level of ER:PR-B interactions was more prognostic for relapse. ER levels were not associated with relapse. The outcomes of patients in our cohort, which only included node-positive patients with ER+ breast cancer, are highly dependent on the efficacy of systemic adjuvant treatments, including chemotherapy and endocrine therapy. Non-compliance with endocrine therapy is well-recognised as being associated with adverse disease outcomes.^52^ Effective adjuvant endocrine therapy is clearly critical in preventing relapse in node-positive ER+ breast cancer.

When stratified by type of endocrine agent taken, the prognostic effect of ER:PR-B interactions was limited to patients on AIs, predominantly in post-menopausal women. The low numbers of patients in the tamoxifen treated group, most of whom were pre-menopausal, may be partially responsible for the lack of prognostic significance of ER:PR-B interactions. A significant test for interaction indicated that ER:PR-B interactions may be predictive of AI efficacy. These findings need to be replicated in prospective randomised controlled trials to determine whether ER:PR-B interactions or PR-B expression by PLA may be used to select for adjuvant endocrine treatment. There are several large phase III trials of cyclin-dependent kinase (CDK) 4/6 inhibitors in combination with endocrine therapy for ER-positive (ER+) early stage breast cancer.^53–55^ ER:PR-B interactions may also serve as predictors of benefit to CDK4/6 inhibitors by identifying patients likely to relapse on standard adjuvant endocrine therapy.

In conclusion, while there is abundant clinical data showing that PR agonists are beneficial in postmenopausal patients with advanced ER+ breast cancer,^56–59^ their use as an adjuvant therapy is not established and trials are in progress.^28^ Several recent pre-clinical studies provide compelling evidence that the key to effective progestogen therapy is the ability of activated PR (specifically PR-B) to reprogram the genomic activity of activated ER. Herein, we describePLA assays which can detect the interaction of ER and PR-B and expression of PR-B in FFPE tissues that could feasibly be automated to facilitate use in diagnostic histopathology laboratories. We further show that assessment of these interactions could have clinical value and propose that measuring the level of ER:PR-B interactions may predict benefit from progestogen treatment and aid patient selection in future randomised clinical trials of progestogens. Quantifying PR-B levels by PLA appears to be a refinement on PR IHC, which may explain why it is more prognostic for relapse in patients on adjuvant tamoxifen. Finally, we find that ER:PR-B interactions are associated with relapse in patients taking adjuvant AIs, suggesting that ER:PR-B interactions may have utility in predicting efficacy of AIs as well as response to progestogen therapy.

## Electronic supplementary material

Supplementary Figure 1

Supplementary Figure 2

Supplementary Figure 3

Supplementary Tables

## Availability of data and materials

To protect patient privacy, all patient data have been de-identified and reported in aggregate. De-identified patient data are available from the authors on request by e-mail.
