# Supplementary figures and images for: Improved relapse-free survival on aromatase inhibitors in breast cancer is associated with interaction between oestrogen receptor-α and progesterone receptor-b
Source: Br J Cancer. 2018 Nov 9;119(11):1316–25. doi: 10.1038/s41416-018-0331-3 (PMC6265321; doi:10.1038/s41416-018-0331-3)

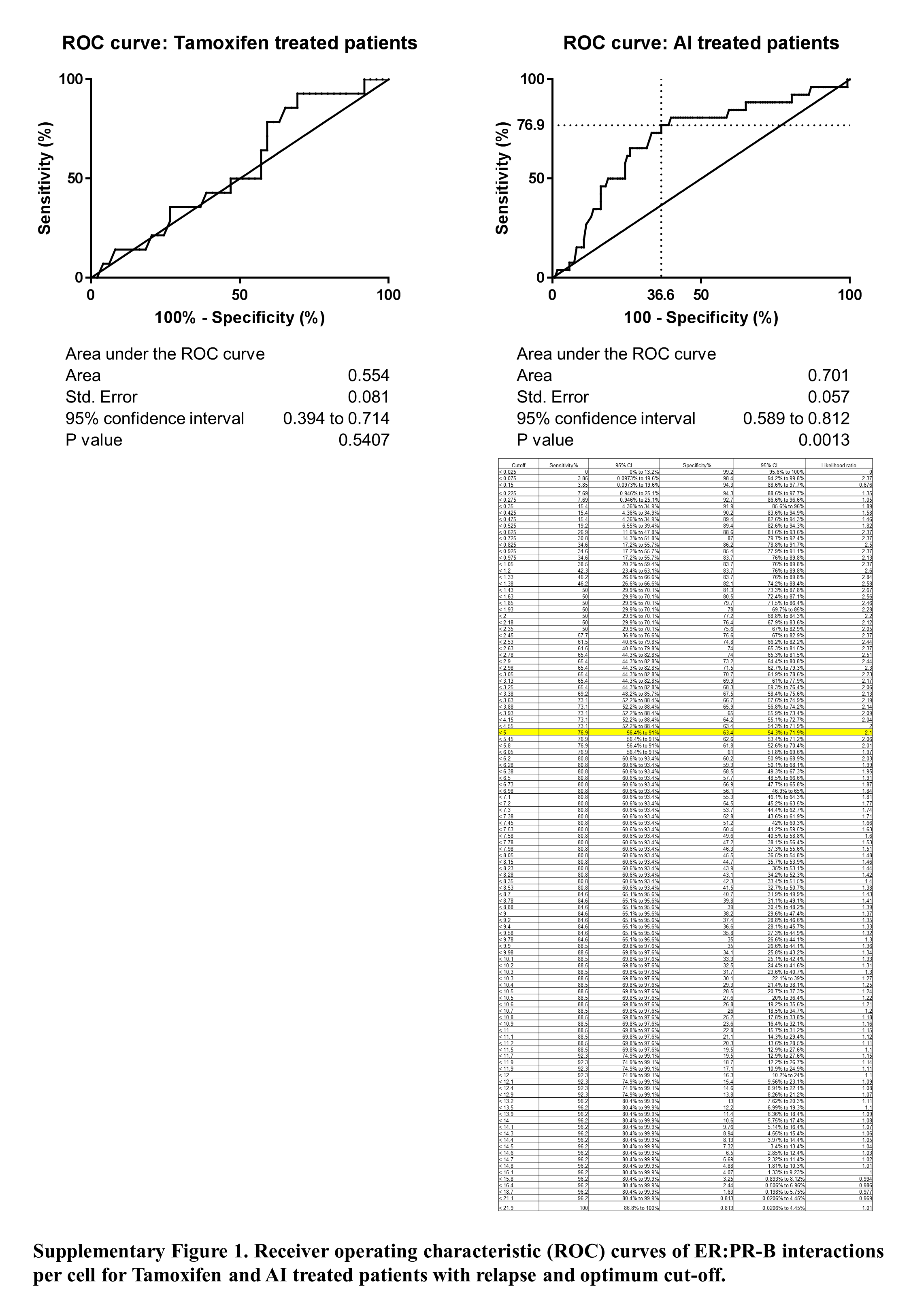

Supplement: Supplementary file 1 — Supplementary Figure 1 [file 41416_2018_331_MOESM1_ESM.tif]

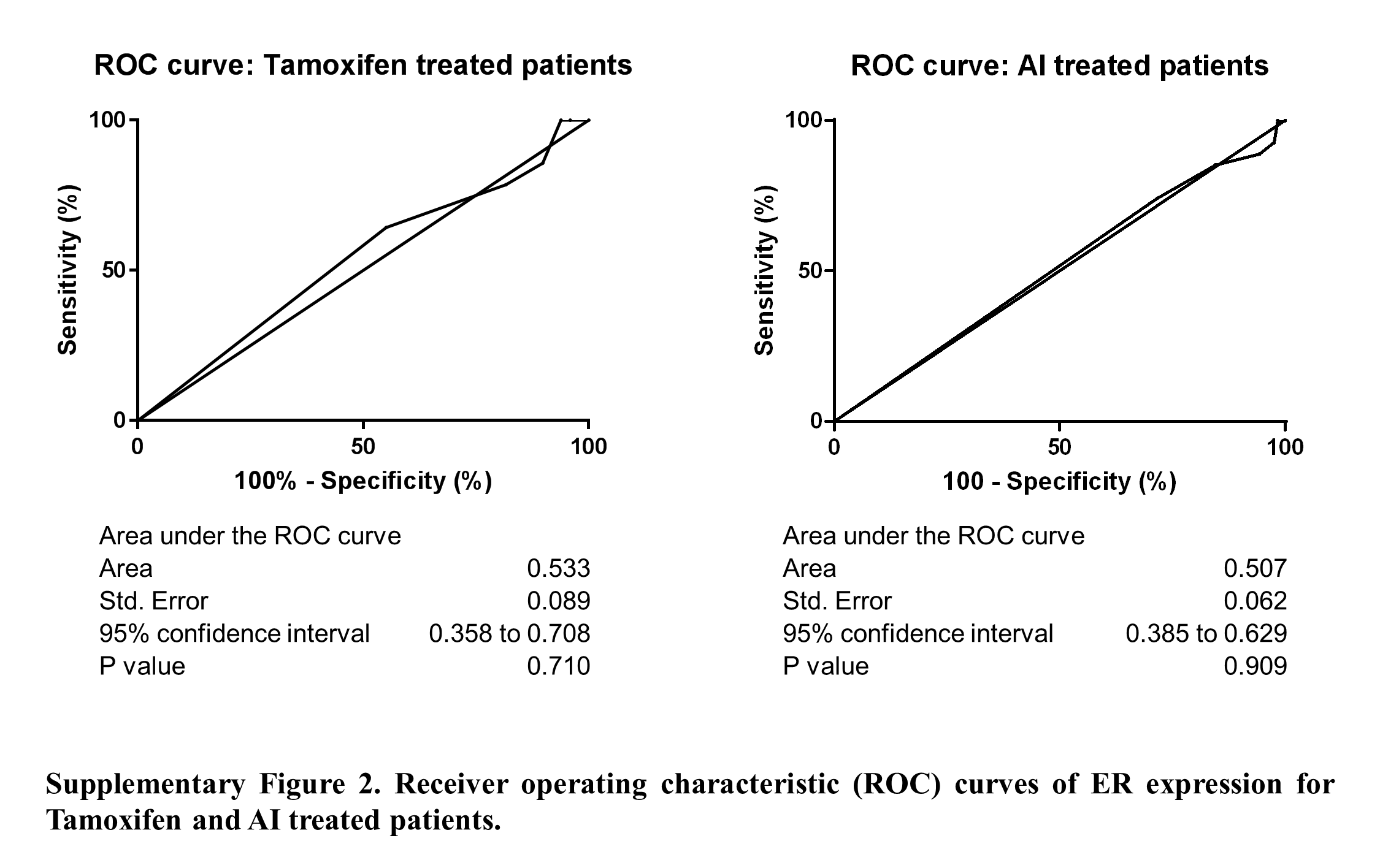

Supplement: Supplementary file 2 — Supplementary Figure 2 [file 41416_2018_331_MOESM2_ESM.tif]

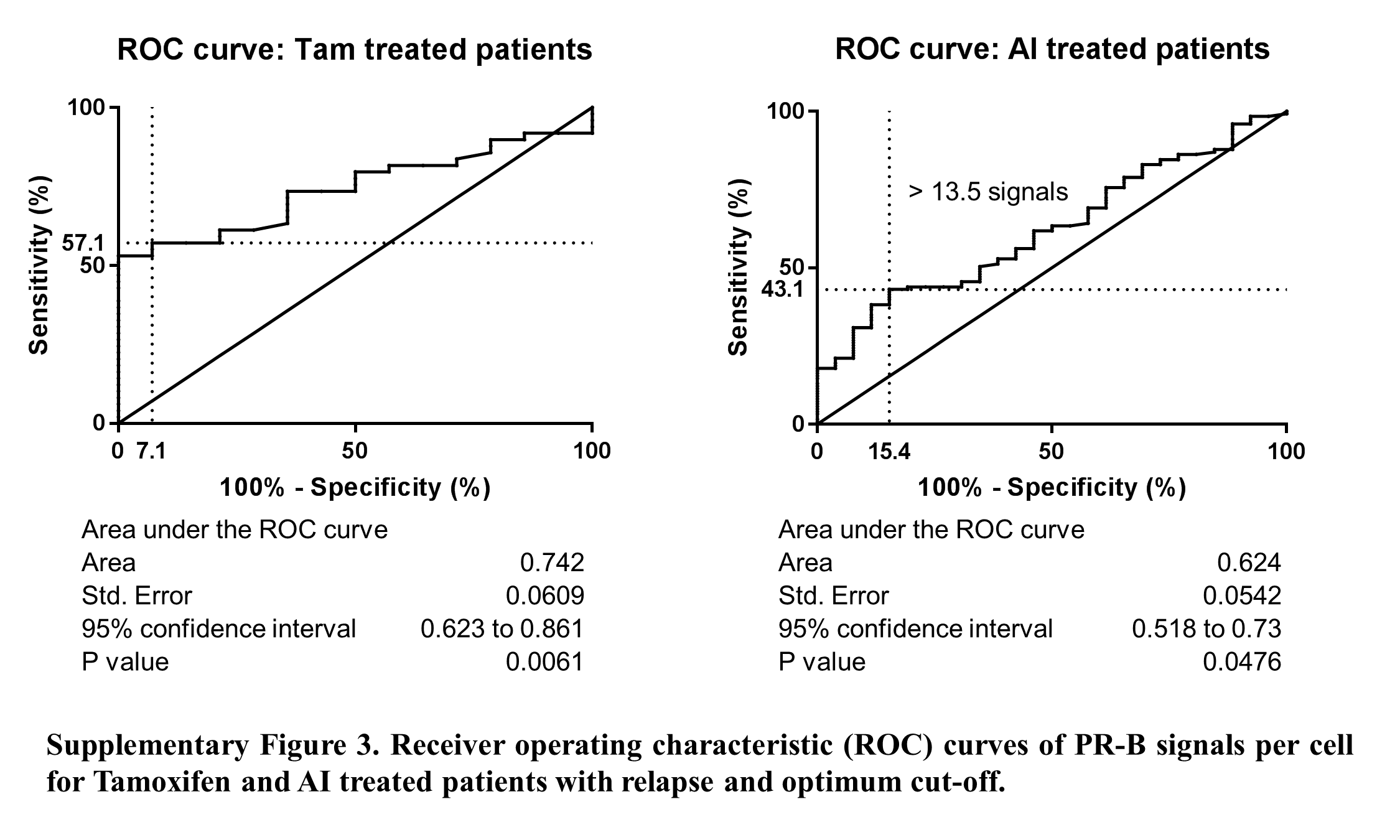

Supplement: Supplementary file 3 — Supplementary Figure 3 [file 41416_2018_331_MOESM3_ESM.tif]
