# Supplementary material for: Improved relapse-free survival on aromatase inhibitors in breast cancer is associated with interaction between oestrogen receptor-α and progesterone receptor-b
Source: Br J Cancer. 2018 Nov 9;119(11):1316–25. doi: 10.1038/s41416-018-0331-3 (PMC6265321; doi:10.1038/s41416-018-0331-3)
Supplement: Supplementary file 4 — Supplementary Tables [file 41416_2018_331_MOESM4_ESM.docx]

| **Variable** | **Number (%)** |
| --- | --- |
| **Age**  < 40  ≥ 40  (Median Age 54) | 23 (10.0)  206 (90.0) |
| **Post-menopausal**  Yes  No | 163 (71.2)  66 (28.8) |
| **T-stage**  pT1  pT2  pT3  pT4 | 48 (21.0)  100 (43.7)  68 (29.7)  13 (5.7) |
| **N-stage**  pN1  pN2  pN3 | 139 (60.7)  64 (27.9)  26 (11.4) |
| **Grade**  1  2  3 | 25 (10.9)  143 (62.4)  61 (26.6) |
| **Mitotic score**  1  2  3 | 142 (62.0)  54 (23.6)  32 (14.0) |
| **Histology**  NST  Lobular  Other | 140 (61.1)  38 (16.6)  51 (22.3) |
| **Multiple tumours**  No  Yes | 164 (71.6)  65 (28.4) |
| **Mastectomy**  **Conservation** | 147 (64.2)  82 (35.8) |
| **Axillary clearance**  **Sentinel nodes** | 208 (90.8)  21 (9.2) |
| **Chemotherapy**  **No chemotherapy**  Unknown | 197 (86.0)  31 (13.5)  1 (0.4) |
| **Radiation**  **No radiation**  Unknown | 187 (81.7)  39 (17.0)  3 (1.3) |
| **Adjuvant endocrine therapy**  Yes  No  Unknown | 217 (94.8)  11 (4.8)  1 (0.4) |
| **Class hormonal therapy**  A.I.  Tamoxifen  Unknown | 151 (69.6)  63 (29.0)  3 (1.4) |
| **PR expression**  Positive  Negative  Unknown | 183 (79.9)  45 (19.7)  1 (0.4) |

**Table S1. Clinical and pathological variables of 229 included patients.**

| **Variable** | **Tamoxifen (%)** | **A.I. (%)** | ***p*** |
| --- | --- | --- | --- |
| **Age**  < 40  ≥ 40 | 15 (23.8)  48 (76.2) | 5 (3.3)  146 (96.7) | **<0.001** |
| **Post-menopausal**  Yes  No | 29 (46.0)  34 (54.0) | 125 (82.8)  26 (17.2) | **<0.001** |
| **T-stage**  pT1  pT2  pT3  pT4 | 19 (30.2)  23 (36.5)  20 (31.7)  1 (1.6) | 28 (18.5)  69 (45.7)  43 (28.5)  11 (7.3) | **0.020** |
| **N-stage**  pN1  pN2  pN3 | 41 (65.1)  14 (22.2)  8 (12.7) | 91 (60.3)  43 (28.5)  17 (11.3) | 0.746 |
| **Grade**  1  2  3 | 7 (11.1)  42 (66.7)  14 (22.2) | 17 (11.3)  94 (62.3)  40 (26.5) | 0.386 |
| **Mitotic score**  1  2  3 | 42 (66.7)  14 (22.2)  7 (11.1) | 93 (62.0)  35 (23.3)  22 (14.7) | 0.449 |
| **Histology**  NST  Lobular  Other | 45 (71.4)  10 (15.9)  8 (12.7) | 83 (55.0)  27 (17.9)  41 (27.2) | **0.040** |
| **Multiple tumours**  No  Yes | 44 (69.8)  19 (30.2) | 110 (72.8)  41 (27.2) | 0.387 |
| **Mastectomy**  **Conservation** | 18 (28.6)  45 (71.4) | 61 (40.4)  90 (59.6) | 0.121 |
| **Axillary clearance**  **Sentinel nodes** | 59 (93.7)  4 (6.3) | 135 (89.4)  16 (10.6) | 0.443 |
| **Chemotherapy**  **No chemotherapy** | 58 (92.1)  5 (7.9) | 128 (84.8)  23 (15.2) | 0.185 |
| **Radiation**  **No radiation** | 53 (85.5)  9 (14.5) | 124 (83.2)  25 (16.8) | 0.838 |
| **PR expression**  Positive  Negative | 55 (87.3)  8 (12.7) | 117 (78.0)  33 (22.0) | 0.131 |
| **ER:PR-B interactions**  High  Low | 26 (41.3)  37 (58.7) | 84 (56.4)  65 (43.6) | 0.051 |

**Table S2. Associations between Tamoxifen and Aromatase inhibitor treated patients and clinicopathological variables.**

Abbreviations: PR-, progesterone receptor negative; PR+, progesterone receptor positive; pT, pathologic tumour; pN, pathologic node; A.I., aromatase inhibitor.

*p* values are the result of the two-sided Fisher’s exact test for 2 x 2 tables. For 2 x 3 and 2 x 4 tables *p* values are the result of the Cochran-Armitage test for trend, except for Histology, which is a two-sided Fisher’s exact test. Bold indicates significant *p* values.

| **Endocrine agent** | **PR expression** | **Relapse (%)** | **HR** | **95% CI** | ***p* value** |
| --- | --- | --- | --- | --- | --- |
| **Tamoxifen** | Negative  Positive | 4 (50.0)  10 (18.2) | 2.695 | 0.827 – 8.772 | 0.100 |
| **Aromatase inhibitor** | Negative  Positive | 8 (24.2)  19 (16.2) | 1.773 | 0.775 – 4.049 | 0.175 |
| **Test for interaction** |  |  |  |  | 0.591 |

**Table S3. Cox regression analysis of PR expression influencing relapse-free survival stratified by adjuvant endocrine agent class taken.** Percentages refer to the number of patients with negative or positive PR expression that had relapsed on endocrine treatment.
